# A nomogram to predict HER2 status in breast cancer patients with HER2-borderline disease as determined via immunohistochemistry

**DOI:** 10.18632/oncotarget.19313

**Published:** 2017-07-17

**Authors:** Qianqian Guo, Kai Chen, Xiaojie Lin, Yi Su, Rui Xu, Yan Dai, Chang Qiu, Xue Song, Siying Mao, Qianjun Chen

**Affiliations:** ^1^ Department of Mammary Disease, Guangdong Provincial Hospital of Chinese Medicine, Guangzhou, Guangdong, P.R. China; ^2^ Guangdong Provincial Key Laboratory of Malignant Tumor Epigenetics and Gene Regulation, Sun Yat-sen Memorial Hospital, Sun Yat-sen University, Guangzhou, Guangdong, P.R. China; ^3^ Breast Tumor Center, Sun Yat-sen Memorial Hospital, Sun Yat-sen University, Guangzhou, Guangdong, P.R. China; ^4^ Department of Intensive Care, Foshan Hospital of Traditional Chinese Medicine, Guangzhou, Guangdong, P.R. China

**Keywords:** breast cancer, HER2 status, IHC, calibration, nomogram

## Abstract

This study aimed to develop a nomogram to predict fluorescence *in situ* hybridization (FISH) assay results for HER2-borderline breast cancer as determined via immunohistochemistry (IHC) among patients in China. We reviewed a database of breast cancer patients diagnosed between January 2007 and April 2013 at our institutions. We used logistic regression to develop a nomogram and we used receiver operating characteristic curve analysis and calibration plots to validate our nomogram. In total, 1138, 301 and 344 patients had IHC-determined HER2-negative, HER2-borderline and HER2-positive disease, respectively. Within the training cohort, univariate and multivariate analyses suggested that estrogen receptor (ER) status, progesterone receptor (PR) status and tumor grade were significantly associated with HER2 status (P<0.01). A nomogram was developed and the AUCs for the training and validation cohorts were 0.795 and 0.749, respectively. The calibration plots suggested that the model was well calibrated. This new nomogram can be used to predict HER2 status in HER2-borderline breast cancer patients and will be particularly helpful to resource-limited countries.

## INTRODUCTION

Globally, breast cancer is the most frequently diagnosed cancer and is the leading cause of cancer-related death among women. It is known that breast cancer is not a single disease; gene expression profiling via microarray analysis according to the mRNA expression levels of specific genes has provided a new method for classifying breast tumors into at least five distinct subtypes: luminal A, luminal B, normal breast-like, HER2-positive and basal-like [1, 2]. Amplification of the HER2/neu gene, resulting in over-expression of this receptor, is found in 20-25% of human breast cancers [3, 4]. Determination of HER2 over-expression in breast carcinomas has become important in clinical practice, with the advent of anti-HER2 therapy as demonstrated in clinical trials [5–7], such as the BCIRG 006, NSABP B31/N9831 and HEAR trials. All these trials have shown that trastuzumab can be beneficial to HER2-positive breast cancer patients. Therefore, the HER2 status is crucial for the guidance of treatment decisions involving the use of trastuzumab, and measurement of the HER2 status is becoming a standard recommendation in the pretreatment work-up of patients with invasive breast cancer.

Before starting anti-HER2 therapy, physicians must be sure of the accuracy of the test results that show HER2 over-expression [8–11]. The expression of the HER2 protein is determined via immunohistochemistry (IHC) in routine practice due to the ease of performance and low cost of IHC [12]. IHC is widely used to detect the expression of HER2 protein and is the preferred method for genetic screening and testing [13–16]. In some tumors, it is difficult to differentiate between 1+ and 2+ or between 2+ and 3+ HER2 expression scores. Hoang [17] previously reported low interobserver reproducibility for distinguishing cases with 2+ HER2 expression from cases with 3+ HER2 expression. Thus, in current clinical practice, the FISH assay is still considered to be the gold standard technique for evaluation of the HER2 status [18–23].

The American Society of Clinical Oncology/College of American Pathologists (ASCO/CAP)[24] also recommend that if results are equivocal, reflex testing should be performed using an alternative assay (IHC or ISH). Because in many laboratories around the world, FISH is the first line test and IHC is the reflex test. However, in addition to the well-known socioeconomic disparities within China [25], substantial regional disparities exist, generally leading to insufficient financial resources and health-care staff in undeveloped regions. IHC is still the first step in HER2 detection. The cost of medical services in China (including surgery and nursing) is very low compared to that in other countries; for example, surgery fees for a mastectomy in Shanghai are $360. Although the FISH assay only costs approximately $300, this cost is an economic burden for poor people. Therefore, we hypothesized that a predictive model could be developed to predict the results of the FISH assay for patients with HER2-borderline disease as determined via IHC. For patients of low socioeconomic status who cannot afford the FISH assay and/or trastuzumab therapy, our model will be helpful in predicting the results of the FISH assay and in providing clarifying information to determine the appropriate treatment options such as chemotherapy and/or endocrine therapy.

In China [26], the incidence of breast cancer among women has increased every year from 2000 to 2011, and this disease has become the leading cause of cancer-related death among women younger than 45 years old. In fact, health professionals in China have long been a group with low income levels [27], and China's per capita income is lower than that of the United States. Therefore, it is necessary to produce a predictive model for the results of the FISH assay in HER2-borderline breast cancer patients.

## RESULTS

### Clinicopathological characteristics of the study population

A total of 1783 female breast cancer patients were included, and the study cohort had a median age of 50 years (range 25-101 years). This study included 1138 (63.82%), 301 (16.89%) and 344 (19.29%) patients determined to be HER2-negative, HER2-borderline and HER2-positive, respectively. Thus, 301 patients had a HER2 score of 2+, of which one case was still not confirmed based on the result of a FISH assay; 161 of these patients did not undergo a FISH assay. Among the patients with IHC-determined HER2-borderline disease, 96 and 43 had negative and positive results for HER2 status on the FISH assay, respectively. Patients with HER2-negative or HER2-positive disease as determined via IHC represented the training cohort, whereas those with IHC-determined HER2-borderline (2+) disease represented the validation cohort. Table 1 compares the baseline characteristics of the training cohort and the validation cohort.

**Table 1:** Clinical pathological characteristics (training cohort and validation cohort)

Characteristic	Training cohort (n=1482)	Validation cohort (n=139)	*P*
**Age**			0.508
Median(range)	49(25-87)	51(31-84)	
<50yr_no.(%)	466(31)	65(47)	
>=50yr_no.(%)	1016(69)	74(53)	
**ER**			0.05
negative_no.(%)	415(28)	22(16)	
positive_no.(%)	1067(72)	117(84)	
**PR**			0.07
negative_no.(%)	449(30)	37(27)	
positive_no.(%)	1033(70)	102(73)	
**Ki67**			0.01
<14%_no.(%)	548(37)	62(45)	
>=14%_no.(%)	934(63)	77(55)	
**T-STAGE**			0.44
T1_no.(%)	683(46)	66(47)	
T2-4_no.(%)	799(54)	72(53)	
**N-STAGE**			0.13
N0_no.(%)	882(60)	79 (57)	
N1_no.(%)	393(27)	45(32)	
N2_no.(%)	124(8)	5(4)	
N3_no.(%)	83(6)	10(7)	
**Grade**			0.22
1_no.(%)	70(5)	6(4)	
2_no.(%)	1058(71)	88(63)	
3_no.(%)	354(24)	44(32)	

### Development of a nomogram

**Figure 1:**
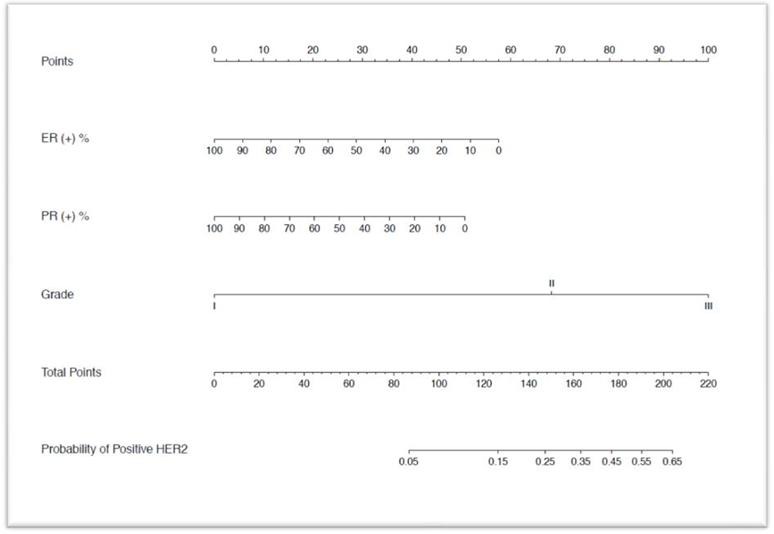
Nomogram to calculate the probability of HER2 positive in breast carcinoma

**Figure 2:**
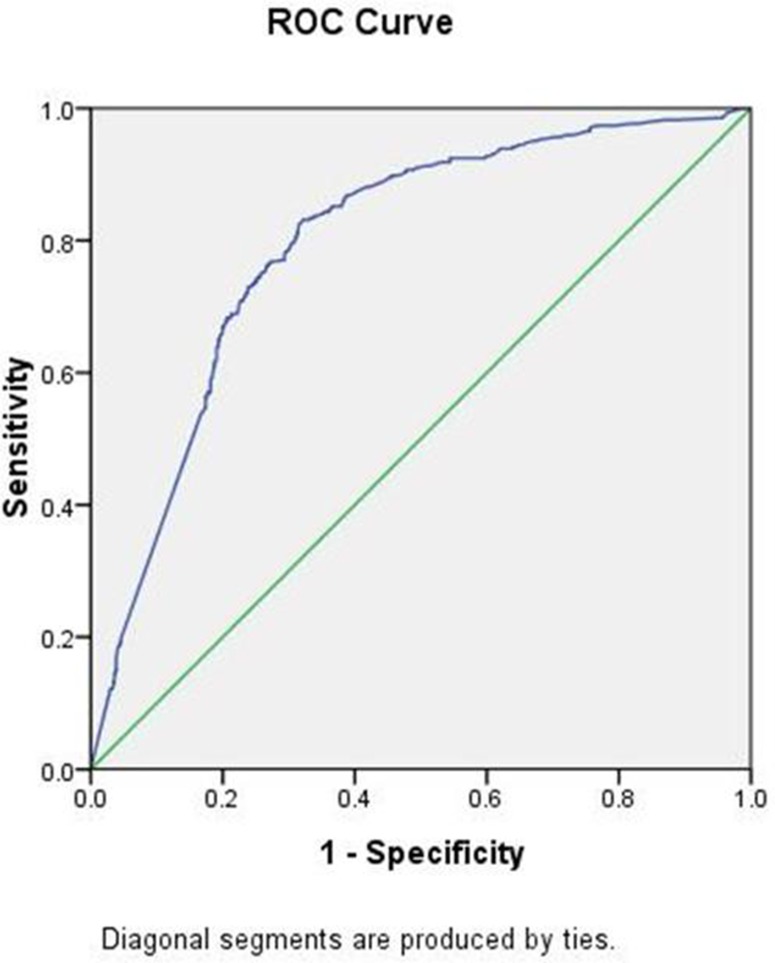

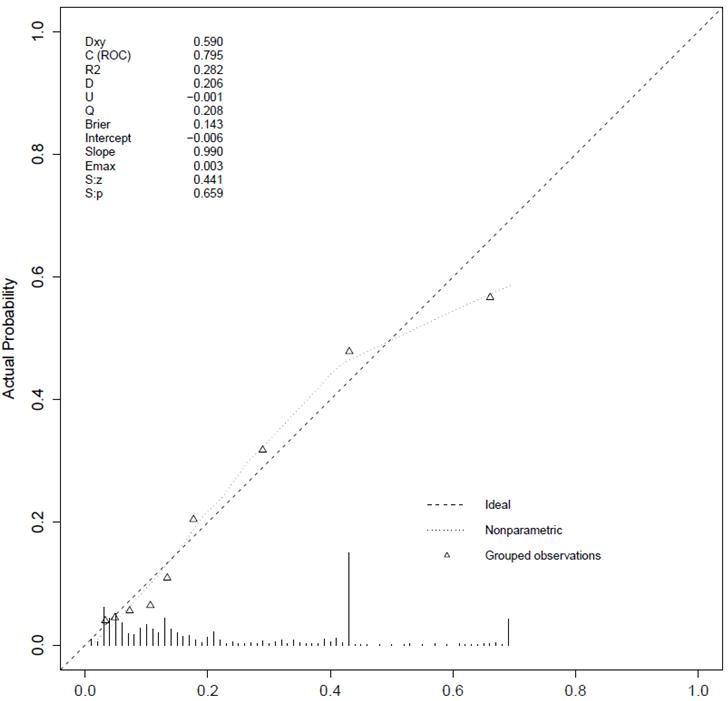
**(a)** ROC curve of the training set. **(b)** Calibration plots of the nomogram validated internally in the training cohort.

**Figure 3:**
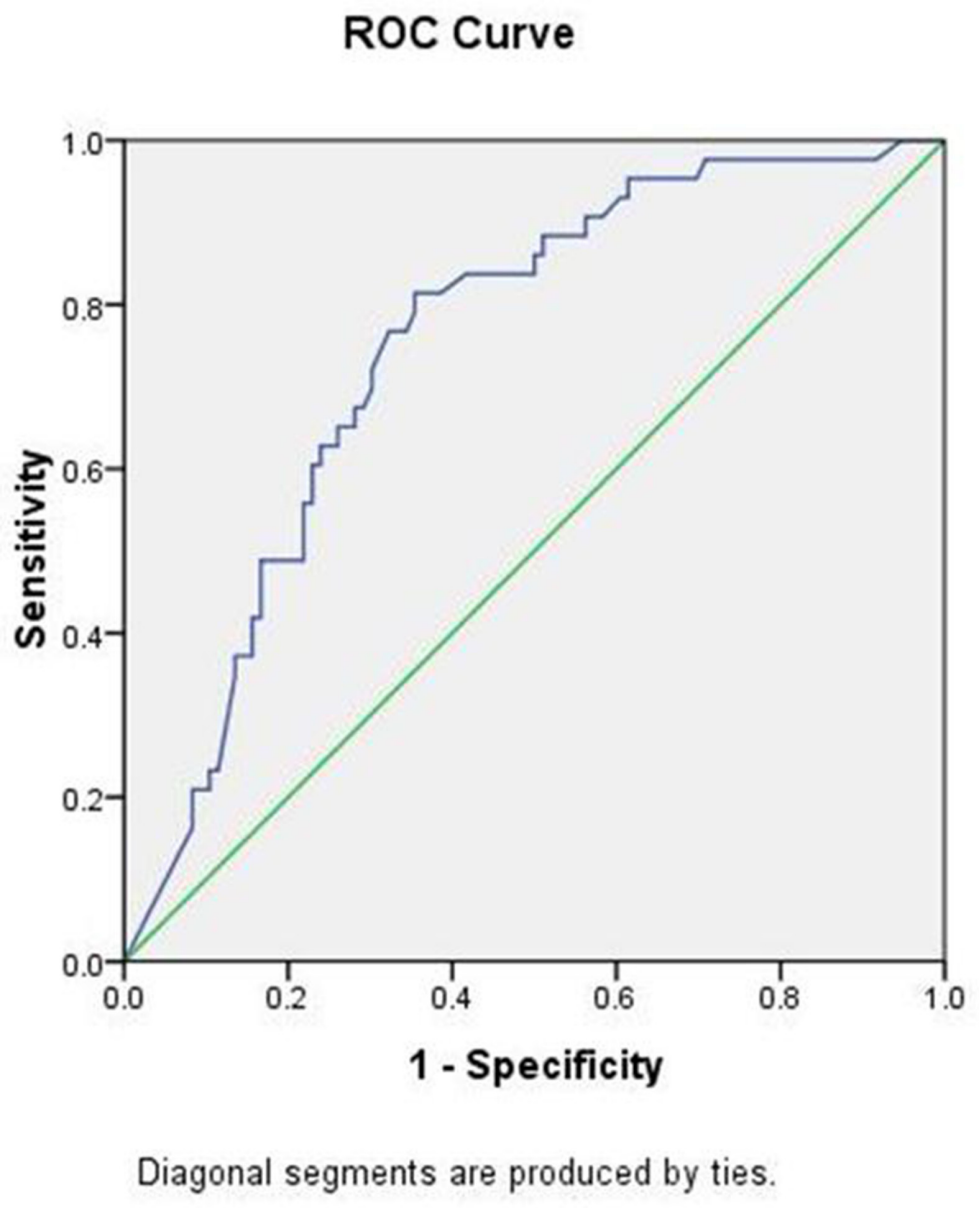

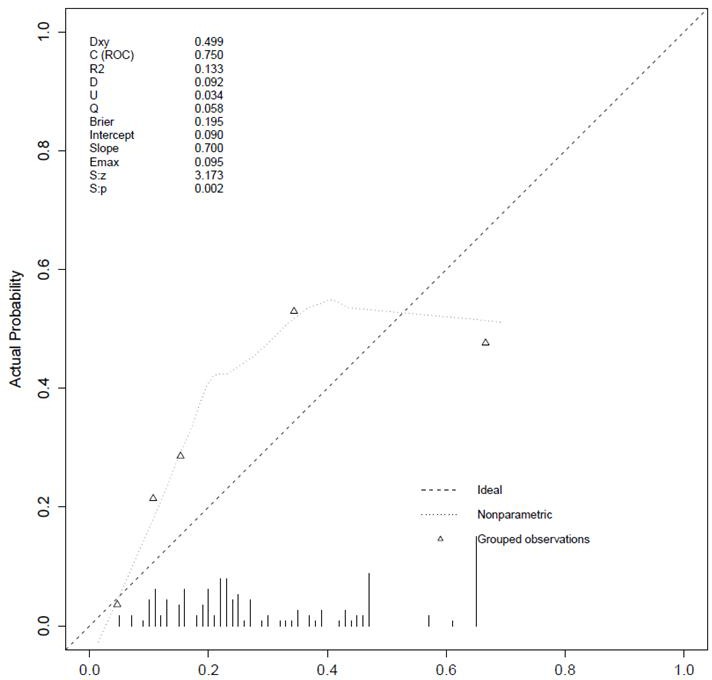
**(a)** ROC curve of the validation set. **(b)** Calibration plots of the nomogram validated internally in the validation cohort.

All variables analysis including age, ER, PR, Ki67, T-stage, N-stage, grade were included in the analysis. The univariate analyses selected ER, PR, N-stage and grade, while in the multivariate analyses revealed ER status, PR status and tumor grade as independent predictors of HER2 status (Table 2). The performance of the nomogram in the validation population was analyzed in terms of discrimination and calibration. A nomogram was developed using multivariate logistic regression analysis including the above predictors (Figure 1). As an internal validation, we performed ROC curve analysis, and the area under the curve (AUC) was 0.795 in the training cohort (Figure 2a). We used ROC curve analysis of the nomogram in our validation cohort for external validation. The AUC for the validation cohort was 0.749 (Figure 3a). The discrimination of the nomogram is satisfactory in both populations with AUC values >0.70. The calibration plots (Figure 2b) revealed that the nomogram was internally well calibrated, with an average estimated error of 2.28%, and the calibration plots revealed that the predicted probability according to the nomogram was slightly higher than the actual probability, with an average estimated error of 10.8% (Figure 3b). The main concern is the P value of Unreliability index, which the two groups were all 1. It is shown that the null hypothesis is not rejected (H0: intercept=0, slope=1), that is, the fitted line coincides with the 45 degree line, which means the prediction is accurate. We used YOUDEN index to determine the cut off value, which with a sensitivity of 81%, specificity of 64%, positive predictive value of 50%, negative predictive value of 88%. Although this result is very good, but may not necessarily acceptable in clinical.

**Table 2:** Univariate and multivariate analysis of risk factors for HER2 character in training cohort

Features	Univariate analysis P value	Multivariate analysis		
		HR	95%CI	P value
age	NS	-	-	NS
ER	<0.01	0.98	0.98-0.99	<0.01
PR	<0.01	0.98	0.98-0.99	<0.01
Ki67	NS	-	-	NS
Grade	<0.01	2.93	2.22-3.88	<0.01
T-STAGE	NS	-	-	NS
N-STAGE	<0.01	-	-	NS

## DISCUSSION

For breast cancer patients who do not undergo the FISH assay, the ability to predict the FISH assay result would be informative for clinical decision making. In this study, we developed a nomogram using a training cohort of patients with IHC-determined HER2-negative or HER2-positive disease. The results of ROC curve analysis suggested that the nomogram has valid discrimination ability, although the calibration plots revealed that the actual predicted deviation and actual probability were slightly greater in the validation cohort than in the training cohort. Our nomogram calculated the predicted probability of a positive FISH assay result. In the absence of further studies, the cut-off value for this nomogram in clinical practice remains unclear. From our perspective, 5% of the positive HER2 status results can be considered as HER2-negative, although these patients still need to be evaluated by a clinician. In reality, clinicians will not act on a 2+ HER2 score in most cases, even if the likelihood of HER2-positive disease is high based on our nomogram. Further, the cost of trastuzumab or other anti-HER2 therapies far outweighs the cost of a single FISH assay. Our nomogram may benefit the detection of a negative HER2 status. In fact, the results of our nomogram should be fully discussed with patients during clinical decision making.

Previous data have shown the relationships between HER2 status and other IHC-determined indexes. An inverse association has been described between HER2 over-expression and the presence of the steroid hormone receptors ER and PR in both clinical correlative studies [28–33] and experimental models [34, 35]. Konecny [36] found that patients with higher levels of HER2 amplification had significantly lower levels of ER/PR than patients with lower levels of HER2 amplification. This finding was consistent with the results of other studies [37, 38]. Our study confirmed that the ER and PR statuses were positively correlated with the HER2 status. Coincidentally, tumor grade was also previously shown to be associated with the HER2 status as determined via IHC alone [12, 39]. HER2 expression was previously found to correlate with a higher nuclear grade but not with the tumor stage [40]; a majority of studies have reported an association between high tumor grade and HER2/neu status [41]. However, none of these studies developed a predictive model incorporating these predictors. Our study is the first to develop a nomogram to perform this prediction.

Clinical studies have confirmed that HER2-positive breast cancer patients have significantly higher risks of recurrence and mortality than HER2-negative breast cancer patients. In Slamon’s study [3], breast cancer patients harboring greater than five copies of HER2 had shorter disease-free survival durations (P=0.015) and overall survival durations (P=0.06) than those without HER2 gene amplification. Several other studies have also shown an association between patient prognosis and the molecular subtype of breast cancer. Millar [42] found a 5-year locoregional recurrence rate of 15% for HER2-enriched tumors, compared to a 1% rate for luminal A tumors. Voduc et al.[43] reported that the HER2-enriched and basal subtypes of breast cancer were associated with an increased risk of local and regional recurrence. Therefore, it is extremely important to know the HER2 status during clinical decision making. However, China has many rural areas and many poor patients who do not have sufficient resources and medical insurance coverage. Even in cities such as Beijing, 8.9% of patients have no access to HER2 testing, and among those who have uncertain IHC results regarding their HER2 status, 10% of patients do not receive a FISH assay [44]. Therefore, we predict that the situation is much worse in rural areas, although no data are currently available. Additionally, trastuzumab is typically not included in national or local reimbursement listings, resulting in prohibitively high out-of-pocket expenses for this drug for many patients [45]. Thus, our prediction model has practical value for patients of low socioeconomic status. For patients who cannot afford the FISH assay and trastuzumab therapy, our model will be helpful for predicting their HER2 status. If an patient with IHC-determined HER2-borderline disease were predicted to be HER2-positive and that patient could not afford trastuzumab, a stronger chemotherapy regimen, e.g., dose-dense AC-T, could be considered as an alternative to TC regimens.

Several limitations of this study should be noted. 1) The small sample size of our study population may have compromised the power of our statistical analyses. For example, the PR showed a lot of original data because of the small sample size problem, which we did not use the residual analysis method to evaluate the abnormal value, so the value still retained which increases the degree of discrete regression relationship to some extent. 2) This is not a multicenter validation study, which may limit the results of the study. 3) Inconsistent detection of Ki-67 may affect its relationship with HER2. 4) Patients receiving neoadjuvant chemotherapy were not included in our study. Therefore, our nomogram is not valid for these patients. 5) All patients in our study cohort had invasive ductal carcinoma. Therefore, it is unclear whether our nomogram can be applied to other pathological types of breast cancer, such as mucinous carcinoma and medullary carcinoma. 6) Internal validation is not sufficient, We still need external data for further verification. 7) In our study, data were not available for several important indexes, e.g., LVI, Allred score for ER status, EIC, P53, and TOPO II status. Our nomogram appears to be oversimplified in that it only takes into account ER status, PR status, and histological grade. Therefore, in this model, every poorly differentiated triple-negative tumor may have the same probability of HER2 positivity as a poorly differentiated carcinoma that is hormone receptor-negative and (actually) HER2-positive. Thus, more studies are needed to determine whether adding the indexes noted above would improve our predictive model.

## MATERIALS AND METHODS

### Patients and tissue specimens

We reviewed an electronic database of breast cancer patients diagnosed between January 2007 and April 2013 at our institutions. The ethics committee of Guangdong Provincial Hospital of Chinese Medicine exempted this retrospective study from full ethics review. Our study included 1783 patients treated for operable breast cancer who were diagnosed at Guangdong Provincial Hospital of Chinese Medicine. The main inclusion criterion was newly confirmed early invasive ductal breast cancer with a pathological stage of I-III without any prior treatment (chemotherapy, hormone therapy or radiotherapy). The exclusion criteria were multifocal or bilateral breast cancer. We collected the relevant information about the patients, including age, ER status, PR status, Ki-67 index, HER2 status, T-stage, N-stage and tumor grade. All of our primary data (de-identified) on the patients included in this study are available upon request.

All patients underwent surgical treatment, and the study samples consisted of postoperative paraffinized specimens. Specimens with a volume fraction of 10% neutral formalin were fixed for 6-48 h; then, the specimens were embedded in paraffin and sectioned into 2-4-μm sections. HE staining confirmed the presence of invasive ductal carcinoma, and the IHC method was used to determine the HER2 status, ER status, PR status, Ki-67 index and tumor grade. If the result of IHC for HER2 was a score of 2+, the FISH assay was performed. Expression levels of standard biomarkers at the time of diagnosis were reviewed in all sections that were subjected to IHC. The ER and PR levels were regarded as positive if at least 1% of tumor nuclei stained positive for the respective marker [46].

The US FDA and ASCO/CAP recommend that HER2 IHC scores of 0 and 1+ be regarded as HER2-negative and that HER2 scores of 3+ be considered as HER2-positive. A case of invasive breast cancer with an HER2 score of 2+ is regarded as HER2-borderline and should be further assessed via a FISH assay, which is considered the gold standard test of HER2 status. The number of HER2 gene amplifications was determined via FISH using an FDA-approved dual-color PathVysion HER2 DNA Probe Kit and a Paraffin Pretreatment Kit. The kit contains a mixture of a spectrum of orange-labeled HER2/neu gene probes and a spectrum of green-labeled centromere controls for chromosome 17. The HER2/CEN-17 ratio was calculated by dividing the total HER2 signal by the total CEN-17 signal. A negative HER2 FISH assay result [18] is defined as a HER2/CEP17 ratio of less than 1.8 or an average of fewer than four copies of the HER2 gene per nucleus. A positive FISH assay result is defined as an elevated HER2 gene copy number (average of >six gene copies/nucleus) or a HER2/CEP17 ratio of greater than 2.2.

### Statistics

Descriptive statistics were obtained for the entire dataset, and all statistical analyses were performed using SPSS version 19.0 software. The Chi-square test was used to compare categorical variables, and the Kolmogorov-Smirnov Z test was used to compare continuous variables. Univariate and multivariate logistic regression analyses were used to determine the independent factors predicting HER2 over-expression. The “Enter” method was used to select variables for the logistic regression analyses. We used the rms package in R software to develop a nomogram based on the results of the logistic regressions. For model validation, we used receiver operating characteristic (ROC) curve analysis and a calibration plot to evaluate the performance of the nomogram. The calibration plot was a graphical representation of the agreement between the observed outcome frequencies and the predicted probabilities, which was used to verify whether or not the model has fitted the data well. All statistical tests were two-sided, and a value of P <0.05 was considered significant.

## CONCLUSION

Our nomogram predicts the results of the FISH assay in breast cancer patients with IHC-determined HER2-borderline disease. In the future, we suggest three directions for further investigation. First, more external validation studies are needed to validate our nomogram. Second, the cost-effectiveness of our model must be evaluated. Third, a randomized controlled trial may be needed to confirm that more intensive adjuvant therapy would provide a survival benefit to patients predicted to have a high risk for positive HER2 status based on the nomogram.
